# Supplementary figures and images for: Impact of Fibrous Microplastic Pollution on Commercial Seafood and Consumer Health: A Review
Source: Animals (Basel). 2023 May 24;13(11):1736. doi: 10.3390/ani13111736 (PMC10252135; doi:10.3390/ani13111736)

Figure S1. Flow chart outlining the criteria for the inclusion of studies in literature review.

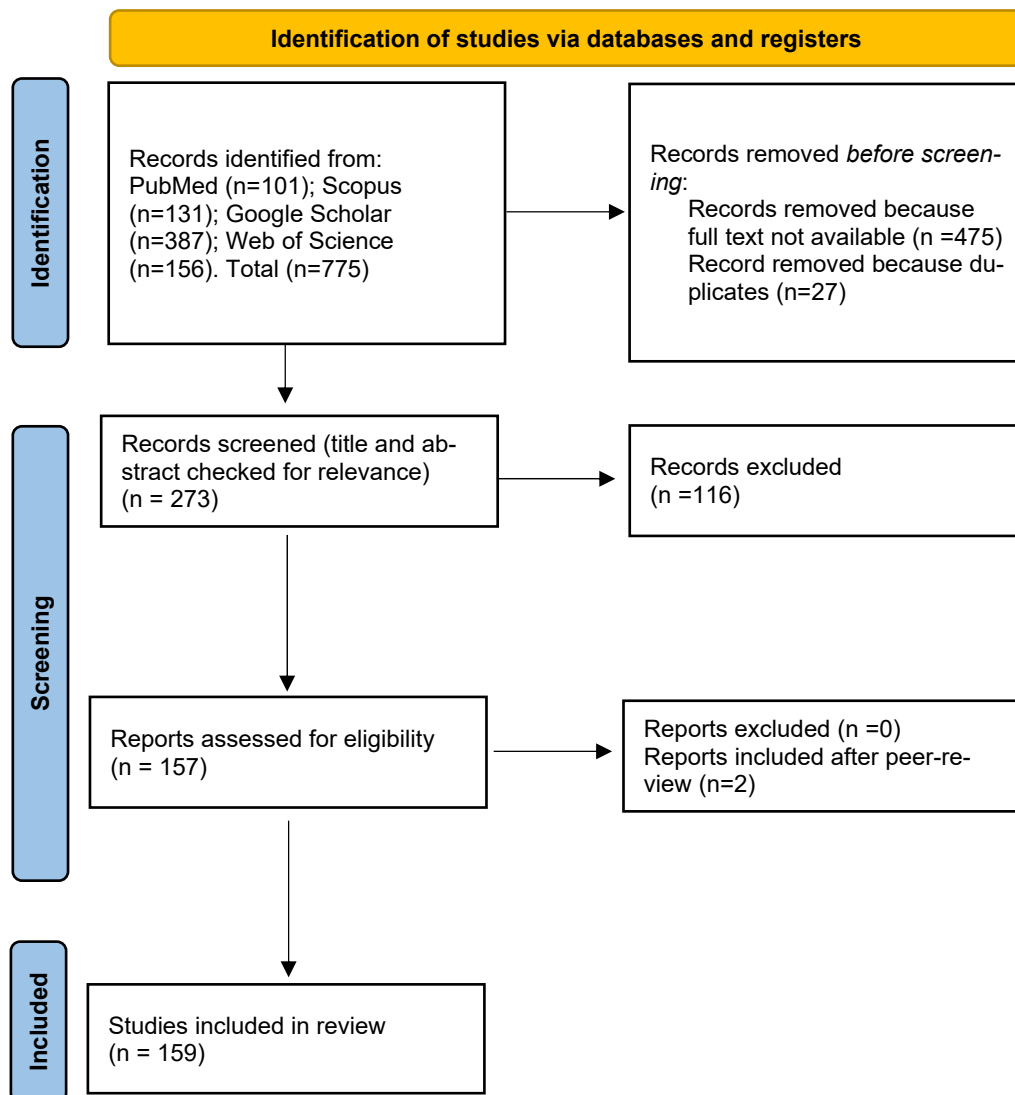

Supplement: Supplementary file 1 [file animals-13-01736-s001.zip › animals-2371542-supplementary.pdf]
